# Ethnic variations in falls and road traffic injuries resulting in hospitalisation or death in Scotland: the Scottish Health and Ethnicity Linkage Study

**DOI:** 10.1016/j.puhe.2020.01.013

**Published:** 2020-05

**Authors:** G. Cézard, L. Gruer, M. Steiner, A. Douglas, C. Davis, D. Buchanan, S.V. Katikireddi, A. Millard, A. Sheikh, R. Bhopal

**Affiliations:** a School of Geography and Sustainable Development, University of St Andrews, St Andrews, UK; b Usher Institute for Population Health Sciences and Informatics, University of Edinburgh, Edinburgh, UK; c Environmental & Occupational Medicine, Section of Population Health, University of Aberdeen, Aberdeen, UK; d Information Services Division (ISD), NHS National Services Scotland, Edinburgh, UK; e MRC Social & Public Health Sciences Unit, Evaluation of Social Interventions Programme, University of Glasgow, Glasgow, UK; f NHS Health Scotland, Directorate of Public Health Science, Glasgow, UK

**Keywords:** Ethnicity, Accidents, Falls, Road traffic injuries, Scotland

## Abstract

**Objectives:**

To investigate ethnic differences in falls and road traffic injuries (RTIs) in Scotland.

**Study design:**

A retrospective cohort of 4.62 million people, linking the Scottish Census 2001, with self-reported ethnicity, to hospitalisation and death records for 2001–2013.

**Methods:**

We selected cases with International Classification of Diseases–10 diagnostic codes for falls and RTIs. Using Poisson regression, age-adjusted risk ratios (RRs, multiplied by 100 as percentages) and 95% confidence intervals (CIs) were calculated by sex for 10 ethnic groups with the White Scottish as reference. We further adjusted for country of birth and socio-economic status (SES).

**Results:**

During about 49 million person-years, there were 275,995 hospitalisations or deaths from fall-related injuries and 43,875 from RTIs. Compared with the White Scottish, RRs for falls were higher in most White and Mixed groups, e.g., White Irish males (RR: 131; 95% CI: 122–140) and Mixed females (126; 112–143), but lower in Pakistani males (72; 64–81) and females (72; 63–82) and African females (79; 63–99). For RTIs, RRs were higher in other White British males (161; 147–176) and females (156; 138–176) and other White males (119; 104–137) and females (143; 121–169) and lower in Pakistani females (74; 57–98). The ethnic variations differed by road user type, with few cases among non-White motorcyclists and non-White female cyclists. The RRs were minimally altered by adjustment for country of birth or SES.

**Conclusion:**

We found important ethnic variations in injuries owing to falls and RTIs, with generally lower risks in non-White groups. Culturally related differences in behaviour offer the most plausible explanation, including variations in alcohol use. The findings do not point to the need for new interventions in Scotland at present. However, as the ethnic mix of each country is unique, other countries could benefit from similar data linkage-based research.

## Highlights

• Ethnic inequalities in injuries are demonstrated in Scotland based on a large sample size and a fine ethnic granularity.
• White minority ethnic groups had the highest risks of fall-related injuries in Scotland.
• Fall-related injuries were the least likely in the Pakistani population.
• Ethnic differences in road traffic injuries varied by the type of road user.
• Ethnic differences in injuries were not explained by socio-economic status or country of birth.

## Introduction

Ethnicity has been defined as ‘the social group a person belongs to and either identifies with or is defined by others as a result of a mix of cultural and other factors including language, diet, religion, ancestry, and physical features’.^1^ These factors have the potential to influence health in many ways and important differences between ethnic and migrant groups have been found for all-cause mortality^2^, ^3^, ^4^ and numerous other health problems.^5^ The Global Burden of Disease Injury and Risk Factors Study reported that in 2013 there were an estimated 56.2 million injuries requiring hospitalisation. The commonest causes were falls (36%) and road traffic injuries (RTIs) (20%).^6^ Of 4.8 million deaths, about 29% were due to RTIs and 11% to falls. In Scotland in 2017, 61% of admissions to hospital for unintentional injury were owing to falls and 5% owing to RTIs.^7^ Whilst the incidence of falls increases markedly in old age, many risk factors are involved including alcohol consumption, which may differ by ethnic group.^8^, ^9^, ^10^, ^11^ RTI rates vary according to mode of transport; intoxication with alcohol or other drugs is also a risk factor. In 2016, 13% of all road deaths in Great Britain involved at least one driver with an alcohol level over the drink-drive limit.^12^ As these factors are socially mediated, it is reasonable to hypothesise that ethnicity could influence the risk of RTI. However, few studies have used whole populations or large representative samples to compare risks of fall-related injuries or RTIs in different ethnic or migrant groups.^13^, ^14^, ^15^ Most have typically involved smaller, often unrepresentative, samples, and self-reported falls rather than injuries.^16^ Several more robust studies of RTIs have been conducted in different countries, often showing clear differences between ethnic groups.^15^^,^^17^, ^18^, ^19^ However, no consistent picture emerges from these studies, probably indicating that the national context affects the patterns found.

The Scottish Health and Ethnicity Linkage Study (SHELS) was established in 2003 as a means of exploring in detail the relationships between ethnic group and health in Scotland. A retrospective cohort was created including 4.62 million people who took part in the Scottish Census 2001 and whose ethnic group was recorded.^20^ By linking the Census results to hospitalisation and death records, it has provided a unique opportunity to explore differences in many health outcomes between the larger ethnic groups in Scotland.^21^, ^22^, ^23^, ^24^, ^25^, ^26^, ^27^, ^28^ The published findings have provided an important evidence base for developing race equality policy in Scotland.^29^ In this article, we examine whether risks of injury or death due to falls or RTIs vary between ethnic groups in Scotland. Large differences could have implications for accident prevention policy.

## Methods

### Data sources

The SHELS methods have been published^20^^,^^30^ and are described in more detail in Supplementary File A. Individual records from the Scottish Census 2001, which included self-defined ethnic group, were linked to the Scottish Community Health Index (CHI), a unique identifier for everyone registered with the National Health Service in Scotland (NHS Scotland). A total of 4.62 million records were successfully linked, 91% of the estimated population of Scotland at that time and at least 85% of each ethnic group (Supplementary File A, Table A1). This enabled confidential linkage of the Census records to health-related datasets that included the CHI identifier.

### Outcomes

The Information Services Division of NHS Scotland provided data on hospitalisations and deaths for 12 years from May 2001 to April 2013. We used International Classification of Diseases 10th Revision codes for injuries due to falls (W00–W19) and RTIs (V01-89), selecting all cases with a relevant code in any of the six diagnosis positions in the hospitalisation records or the 11 cause positions in the death records. Deaths were combined with hospitalisations as the number of deaths were too small to enable separate analyses. All injuries of sufficient severity to at least require a stay in hospital were thus included.

### Ethnic groups

After extensive research and public consultation, the Scottish Census 2001 used an ethnic classification with 14 main groups. These reflect the pattern of immigration to Scotland over the past 70 years. Because of small numbers of cases, we added the Bangladeshi group to the Other South Asian group and combined the Caribbean, Black African, Black Scottish and Other Black groups into an ‘African Origin’ group. We did not report on the ‘All Other Ethnic Groups’ category owing to its heterogeneity. The study therefore examined 10 ethnic groups: White Scottish, Other White British, White Irish, Other White, Any Mixed Background, Chinese, Indian, Pakistani, Other South Asian and African Origin.

### Analysis

An analysis protocol was agreed before the data extraction and followed without modification.^21^ We hypothesised we would find differences of at least 10% in the outcomes between the White Scottish majority group (reference) and minority ethnic groups, which could potentially be of public health importance. We analysed fall-related injuries and RTIs for males and females separately. We also conducted subanalyses of RTIs involving pedestrians (V01–V09), cyclists (V10–V19), motorcyclists (V20-29), and car occupants (V40–V49).

Person-years at risk over 12 years were used as the denominator, adjusted for either death or known departure from NHS Scotland, mostly to elsewhere in the United Kingdom (UK). We calculated age-adjusted rates and risk ratios (RRs) and their 95% confidence intervals (CIs) using Poisson regression with robust variance and stratified by sex. RRs were multiplied by 100 to be interpretable as percentages. We first interpret ethnic differences in our health outcome of interest based on a baseline model adjusted for age and further include additional risk factors to gauge their contribution in explaining the observed ethnic differences.

Following previously described methods,^31^ we used a proxy measure of socio-economic status (SES), combining three indicators which were consistently associated in the same direction with the outcomes across ethnic groups and by sex: the Scottish Index for Multiple Deprivation (an area-based measure), household tenure and a combined measure of highest educational level. Country of birth was categorised as those who were born in the UK or the Republic of Ireland (RoI) compared with those who were born elsewhere. We examined the influence of SES and UK/RoI birth by adjusting for both separately and in combination.

Data were analysed using SAS version 9.4 (SAS Institute Inc, Cary, North Carolina, USA).

### Ethics, security and reporting

The Multicentre Research Ethics Committee for Scotland (REC 13/SS/0225) and the Privacy Advisory Committee of NHS National Services Scotland (PAC 36/13) approved the study. Individual consent for linking these records was not sought. Researchers with appropriate security clearance (GC, MS) carried out the analyses in a secure environment at National Records of Scotland (NRS). An NRS disclosure committee reviewed all outputs before release. For disclosure reasons, numerators and denominators were rounded to the nearest five in the tables; numbers of cases of five or less and their associated results were not released. However, the RRs were calculated using the real number of cases.

In reporting, we complied with the STROBE/RECORD checklist (Supplementary File B).

## Results

### Characteristics of the study population

The ethnic distribution of the SHELS cohort was similar to that of the Scottish Census 2001 population, including 89% White Scottish, 9% other White groups and 2% non-White groups (Table 1). The White groups were on average older than the non-White groups. Around 25% of White Scottish and Pakistani groups had the highest level of educational qualification compared with 40–52% in several other groups. People of African Origin were the most likely to live in the most disadvantaged areas but almost as many lived in the least disadvantaged. Household ownership was highest in the Indian and Pakistani groups. The proportion of people who were born in the UK/RoI was more than 95% for the White Scottish, Irish, and Other British groups, 75% for the Any Mixed Background group and 30–60% for other groups.

**Table 1:** Sociodemographic profile of the linked 2001 Scotland Census population by sex and ethnic group.

Sex and ethnic group	Population	Ethnic distribution	Age at Census	UK/RoI-born^a^	SIMD^b^		Highest qualification (individual)	Highest qualification (household)	Household tenure (owned)
					Most deprived	Least deprived			
	N	*%*	*Mean (SD)*	*%*	*%*	*%*	*%*	*%*	*%*
**MALES**									
White Scottish	1,949,484	88.5	38 (22)	99.1	20.1	19.8	24.4	39.0	68.6
Other White British	160,235	7.3	42 (20)	95.3	8.0	29.6	48.4	61.9	72.3
White Irish	20,341	0.9	45 (20)	98.4	22.2	21.7	36.8	51.4	65.4
Other White	29,944	1.4	36 (21)	30.5	12.4	32.6	49.2	66.2	56.6
Any mixed background	5310	0.2	21 (18)	76.0	19.7	26.1	36.8	59.7	57.1
Indian	6448	0.3	31 (19)	48.4	9.7	38.5	50.9	64.5	72.2
Pakistani	12,929	0.6	27 (19)	58.0	15.8	24.8	26.5	47.5	76.2
Other South Asian	3549	0.2	29 (19)	38.9	24.2	28.3	46.4	58.7	53.7
African origin	3277	0.1	30 (18)	39.9	27.6	22.4	52.4	68.2	43.1
Chinese	6532	0.3	30 (18)	38.4	13.4	38.5	31.8	48.3	69.8
**FEMALES**									
White Scottish	2,138,643	88.6	41 (24)	99.1	21.3	19.2	23.8	37.8	65.2
Other White British	174,748	7.2	44 (21)	95.0	8.2	28.9	40.8	58.5	69.5
White Irish	23,162	1.0	49 (21)	98.6	20.1	22.8	38.0	53.4	64.1
Other White	35,711	1.5	37 (21)	26.4	10.8	33.4	51.3	68.7	58.7
Any mixed background	5799	0.2	24 (20)	74.5	18.7	27.3	37.8	60.2	55.8
Indian	5888	0.2	30 (19)	51.1	9.6	39.0	40.7	61.9	73.6
Pakistani	12,702	0.5	26 (18)	60.5	15.4	24.6	22.8	47.1	75.9
Other South Asian	2963	0.1	29 (20)	44.5	22.0	28.9	36.7	55.2	54.5
African origin	3056	0.1	30 (18)	42.1	27.5	24.5	46.1	67.6	45.1
Chinese	6672	0.3	31 (18)	33.9	12.1	39.1	33.3	50.3	70.1

a UK/RoI-born = Born in the UK or Republic of Ireland.

b SIMD = Scottish Index of Multiple Deprivation.

### Hospitalisations and deaths

During 12 years of follow-up and about 49 million person-years at risk, there were 275,995 hospitalisations and deaths due to falls and 43,865 due to RTIs. The RTIs included 17,965 car occupants, 10,630 cyclists, 7375 pedestrians and 6575 motorcyclists.

### Falls

Table 2 shows ethnic differences in hospitalisations and deaths due to falls. With 95% CIs that did not overlap with the reference value 100, age-adjusted RRs were higher for males in the White Irish (131) and Any Mixed Background (124) groups and lower in the Pakistani group (72). For females, RRs were higher for the Other White British (114), White Irish (118), Other White (110), and Any Mixed Background (126) groups and lower for the Pakistani (72) and African Origin (79) groups. Adjustment for UK/RoI birth and SES either separately or combined resulted in small and inconsistent changes in the RRs in either direction.

**Table 2:** Age-adjusted rates per 100,000 person-years at risk (PY) and risk ratios (RRs) for hospitalisations and deaths owing to falls (W00–W19) by sex and ethnic group. RRs are adjusted for age and additionally for socio-economic status (SES), born in the UK/Ireland (UK/RoI-born) and both, with 95% CIs.

Sex and ethnic group	Cases	PY	Rates/100,000 PY	RR (95% CI) Age	RR (95% CI) Age + SES	RR (95% CI) Age + UK/RoI-born	RR (95% CI) Age + SES + UK/RoI-born
**MALES**							
White Scottish	123,410	21,179,755	583	100	100	100	100
Other White British	6595	1,571,080	581	102 (97, 107)	107 (101, 112)	99 (94, 104)	103 (98, 109)
White Irish	1315	202,190	743	131 (122, 140)	127 (118, 136)	126 (117, 135)	122 (114, 132)
Other White	1035	278,515	579	102 (95, 110)	103 (96, 111)	105 (97, 113)	106 (98, 114)
Any mixed background	275	56,265	707	124 (110, 141)	121 (107, 138)	122 (107, 138)	119 (105, 135)
Indian	205	65,945	570	100 (88, 115)	107 (93, 122)	102 (89, 117)	108 (95, 124)
Pakistani	335	146,430	411	72 (64, 81)	75 (67, 84)	73 (65, 82)	76 (67, 85)
Other South Asian	105	35,500	517	91 (76, 109)	89 (75, 106)	92 (77, 110)	90 (76, 107)
African origin	95	32,160	539	95 (77, 117)	89 (72, 110)	96 (78, 118)	90 (72, 111)
Chinese	120	68,685	485	85 (73, 100)	88 (75, 103)	88 (75, 103)	90 (77, 105)
**FEMALES**							
White Scottish	130,075	22,581,190	576	100	100	100	100
Other White British	8745	1,644,435	638	114 (108, 120)	115 (109, 121)	111 (105, 117)	113 (107, 119)
White Irish	1600	216,905	660	118 (110, 125)	116 (109, 124)	114 (109, 122)	114 (106, 122)
Other White	1310	319,915	617	110 (103, 117)	110 (103, 118)	112 (105, 120)	112 (105, 120)
Any mixed background	210	59,970	710	126 (112, 143)	124 (109, 140)	124 (110, 140)	122 (108, 138)
Indian	110	59,925	498	89 (75, 105)	90 (77, 107)	90 (76, 107)	92 (78, 108)
Pakistani	200	143,940	402	72 (63, 82)	73 (64, 83)	72 (64, 82)	74 (65, 84)
Other South Asian	70	28,610	558	99 (80, 123)	99 (80, 123)	99 (80, 124)	99 (80, 123)
African origin	60	28,590	442	79 (63, 99)	77 (62, 97)	79 (63, 99)	77 (62, 97)
Chinese	125	68,010	577	103 (82, 129)	104 (83, 131)	105 (84, 132)	107 (85, 134)

CI, confidence intervals.

### Road traffic injuries

Table 3 shows ethnic differences in hospitalisations and deaths for all RTIs combined. With 95% CIs that did not overlap with the reference value, age-adjusted RRs were higher for males in the Other White British (161) and Other White (119) groups. Among females, RRs were higher for the Other White British (156) and Other White (143) groups and lower for Pakistani (74) groups. Adjusting for UK/RoI birth and SES either separately or combined resulted in small and inconsistent changes in either direction.

**Table 3:** Age-adjusted rates per 100,000 person-years at risk (PY) and risk ratios (RRs) for hospitalisations and deaths owing to road traffic injuries (V00–V89) by sex and ethnic group. RRs are adjusted for age and additionally for socio-economic status (SES), born in the UK/Ireland (UK/RoI-born) and both, with 95% CIs.

Sex and ethnic group	Cases	PY	Rates/100,000 PY	RR (95% CI) Age	RR (95% CI) Age + SES	RR (95% CI) Age + UK/RoI-born	RR (95% CI) Age + SES + UK/RoI-born
**MALES**							
White Scottish	26,350	21,179,755	124	100	100	100	100
Other White British	1950	1,571,080	195	161 (147, 176)	151 (138, 165)	163 (149, 179)	153 (139, 167)
White Irish	155	202,190	110	91 (77, 107)	93 (79, 110)	93 (78, 110)	94 (80, 112)
Other White	270	278,515	144	119 (104, 137)	117 (101, 134)	117 (101, 135)	115 (100, 132)
Any mixed background	60	56,265	100	82 (65, 105)	83 (65, 106)	83 (65, 106)	84 (66, 107)
Indian	40	65,945	96	79 (59, 105)	75 (57, 100)	79 (59, 105)	75 (57, 100)
Pakistani	115	146,430	110	90 (75, 108)	91 (76, 109)	90 (75, 108)	91 (76, 109)
Other South Asian	40	35,500	153	126 (95, 168)	129 (97, 171)	125 (94, 167)	128 (96, 170)
African origin	20	32,160	92	76 (52, 112)	80 (54, 118)	75 (51, 111)	80 (54, 118)
Chinese	35	68,685	119	98 (73, 133)	98 (72, 132)	97 (72, 132)	97 (72, 131)
**FEMALES**							
White Scottish	13,170	22,581,190	58	100	100	100	100
Other White British	1230	1,644,435	89	156 (138, 176)	141 (125, 160)	163 (144, 185)	146 (128, 165)
White Irish	90	216,905	51	90 (72, 112)	91 (74, 114)	95 (76, 118)	95 (76, 118)
Other White	195	319,915	81	143 (121, 169)	133 (112, 157)	136 (114, 161)	128 (108, 152)
Any mixed background	25	59,970	41	73 (50, 105)	73 (51, 106)	75 (52, 108)	75 (52, 108)
Indian	20	59,925	53	93 (62, 140)	91 (60, 137)	91 (61, 137)	90 (59, 135)
Pakistani	50	143,940	42	74 (57, 98)	79 (60, 103)	74 (57, 97)	79 (60, 103)
Other South Asian	15	28,610	56	98 (60, 160)	97 (60, 159)	97 (60, 159)	97 (60, 159)
African origin	15	28,590	60	105 (65, 170)	111 (69, 180)	103 (63, 167)	110 (68, 178)
Chinese	20	68,010	66	115 (78, 170)	118 (80, 174)	111 (75, 164)	115 (78, 170)

CI, confidence intervals.

When subgrouped into car occupants, cyclists, pedestrians and motorcyclists, there were typically fewer than 50 events in the non-White ethnic groups, including almost none among female cyclists and motorcyclists (Supplementary File C, Tables C1-4). Among the other ethnic groups, there were contrasting patterns. The Other White British group had higher RRs for car occupants (males 132; females 124), cyclists (males 195; females 245), and male motorcyclists (242) but lower for pedestrians (males 79; females 71). The Other White group had higher RRs for female car occupants (128), cyclists (males 138, females 281) and male motorcyclists (199) but lower for female pedestrians (54). Pakistani males had higher RRs for car occupants (135) and lower for cyclists (56). Adjusting for UK/RoI birth or SES made very little difference.

## Discussion

We found unexpectedly large differences between ethnic groups in Scotland in risks of hospitalisation and death due to falls and RTIs over a 12-year period. Compared with the White Scottish population, age-adjusted risks for falls were higher for White Irish and Mixed males, and for Other White British, White Irish, Other White, and Mixed females; they were lower for Pakistani males and females and African females. For RTIs overall, risks were higher for Other White British and Other White males and females but lower for Pakistani females. There were differing patterns for RTIs of car occupants, cyclists, pedestrians and motorcyclists, with virtually no recorded cases among non-White female cyclists and motorcyclists. Adjustment for SES or UK/RoI birth had little effect on the observed differences.

### Strengths and limitations

The strengths and limitations of the SHELS methods have been discussed in detail.^3^^,^^26^ Their strengths include using a national census-based retrospective cohort of 4.62 million people with reliable recording of self-reported ethnicity, SES and country of birth. By linking the cohort to 12 years of hospitalisation and death data, we had enough cases to compare 10 ethnic groups, including four distinct White groups. However, the numbers of cases in some ethnic groups were too small to be analysed or CIs were very wide, particularly for transport subgroups. We combined hospitalisations and deaths as there were too few deaths to analyse separately. Limiting the analysis to hospitalisations was not part of our analysis plan but given the relatively small proportion of deaths, we think it unlikely this would have altered the findings. We addressed the differing age structure of the ethnic groups by adjustment for age: had there been enough cases to allow it, stratification by age could have revealed age-related differences. Some individuals may have left the UK during the follow-up period which we could not account for, possibly introducing some denominator bias. Individuals in some ethnic groups might be more reluctant than others to attend hospital but as the injuries were severe enough to warrant hospital admission, we think this is unlikely.

### Findings in the light of the published literature

#### Falls

A review of published studies of ethnic and racial differences in falls in older adults found most studies involved small samples, restricted by age group and/or gender and relying on self-reporting falls rather than related injuries.^16^ For example, in a study of mainly elderly women, African-Americans reported 23% fewer falls than Whites.^32^ Elderly Italian-born men in Australia were about 40% less likely to report repeated falls than their Australian-born counterparts.^33^ A study of self-reported falls in the elderly comparing Chinese people in Taiwan, Hong Kong and Australia with White people in Australia found 50–70% lower rates among the Chinese.^34^ In none of the studies was socio-economic status found to be an important associated factor. A national study of deaths owing to falls in the Netherlands found no clear-cut differences between native-born Dutch and four minority ethnic groups.^15^ However, as it only included 62 deaths across all ethnic minorities, the study had limited statistical power. Thus, although these studies are more limited in scope and statistical power, the overall picture of lower risks of falls in some ethnic minorities is consistent with our findings.

#### Road traffic injuries

Most previously published studies of RTIs and ethnicity have reported higher risks among disadvantaged ethnic or indigenous minorities. In the Netherlands, higher risks of death for pedestrian RTIs were found among Turkish, Moroccan, Surinamese and Antillean groups.^15^ In Arizona, compared with non-Hispanic Whites, risks among Hispanics and African-Americans were broadly similar but American Indians had much higher risks of death among both car occupants and pedestrians, often associated with high blood-alcohol levels.^17^ In New Zealand, risks of hospitalisation or death owing to RTIs were 65% higher among Māoris than the European/Other group.^19^ In Australia, Aborigines had overall RTIs rates about twice that of non-Aborigines.^18^ In London, both children and adults defined as Black were more likely than Whites to sustain RTIs, whereas Asians were less likely.^35^ The increased rate among Black children was associated with higher neighbourhood deprivation, poorer local road conditions and a riskier commute to school.^36^^,^^37^ In Israel, Arab children were 36% more likely to be hospitalised for an RTI than Israeli children and 57% more likely to be severely injured.^38^ Poorer road conditions, more careless driving and less supervision of children in Arabic communities were highlighted.

#### Possible causes of the ethnic differences in this study

Although a descriptive study such as this cannot prove causation, it can usefully stimulate hypotheses for further research. In a major review, Davey Smith and colleagues considered the possible causes of apparent health differences between ethnic groups under the following headings: artefactual; migration; socio-economic factors; culture, beliefs and behaviours; racism; biology; and health service use and access.^39^ Given the fall-related injuries and RTIs in this study were fatal or sufficiently severe to require hospitalisation, we can expect their assessment and recording to have been consistent regardless of ethnic group and therefore the observed differences are likely to be real. Adjusting for whether individuals were born outside the UK made little difference to either set of findings, suggesting that recent migration to Scotland was not a major explanatory factor, although a smaller effect cannot be excluded.

We found that adjusting for SES, including the socio-economic profile of place of residence, had little effect on our age-adjusted findings for falls. This is supported by the findings of a review by Todd et al.^40^ who did not find a consistent relationship between SES and falls in the elderly. Adjusting our RTI data for SES also had little effect. As aforementioned, this may appear to contrast with the findings of many other studies where disadvantaged ethnic minorities had higher RTI rates. However, the SES of the larger ethnic minority groups in Scotland is notably different from that of many other countries, typically being characterised by larger than average proportions with high educational attainment and home-ownership (Table 1).

Differences in culture, beliefs and behaviours may provide a more plausible explanation for the differences in fall-related injuries, such as the 70% higher risk among White Irish than Pakistani males or the 40–70% higher risks among the White minority females compared with the Pakistani and African females, even after adjusting for SES. One possible contributor is alcohol, long recognised as a risk factor for falls.^11^^,^^40^ A health survey of ethnic minorities in Glasgow found 91% of Pakistanis reported they did not use alcohol compared with 30% in the general (White) population.^41^ Another SHELS study found that White Irish males and females had risks of alcohol-related diseases 3.1 times and 3.6 times higher than their respective Pakistani counterparts.^22^ Many other factors are associated with falls in the elderly, such as gait problems, vertigo, Parkinson's disease and antiepileptic drug use.^8^ However, there appears to be no evidence currently to relate these to ethnicity. As for RTIs, differential use of modes of transport may largely explain the very low number of cases of cycle or motorbike injuries among Indians and Pakistanis, especially females. UK and Scottish data show much lower cycle use among these minorities.^42^^,^^43^ The lower levels of alcohol consumption by some ethnic minorities may also play a part.

As risks of falls and RTIs were lower in the non-White minorities, racism does not appear to be a plausible contributor to the findings. Given the acute nature of the injuries, and the availability of emergency health services to all in Scotland, it also seems unlikely that differential access to or use of health services would play a part. This is supported by another SHELS analysis showing broadly equitable rates of all-cause hospitalisation between ethnic groups.^26^

### Conclusions

We found unexpected and sometimes large ethnic variations in risks of fall-related injuries and RTIs in Scotland, being typically lower among the non-White groups. Cultural and behavioural differences offer the most plausible explanation but would require further research to be confirmed. Although the findings may not suggest the need for new accident prevention initiatives in Scotland at present, they demonstrate that the risk of unintentional injury can vary considerably between ethnic groups. As every country's ethnic mix is unique and many are undergoing considerable demographic change owing to migration, other countries could benefit from similar data linkage–based research.

## Author statements

### Acknowledgements

The authors thank ISD and National Records of Scotland for their ‘in-house’ technical and advisory contributions to the work. Anne Houghton and Theresa Kirkpatrick gave administrative help. Colin Fischbacher as co-applicant helped to set the study up; Chris Povey was a co-investigator and had the idea of linking the census data to the data held by ISD and performed most of the linkage of census to CHI; Alex Stannard advised on use of census data; David Clark advised on and assisted with linkage to ISD databases; Jamie Pearce advised on data analysis particularly social and economic variables.

### Ethical approval

Ethical approval for the data linkage, security and analyses was granted by the Scottish Multicentre Ethics Committee and the Privacy Advisory Committee of NHS National Services Scotland.

### Funding

The authors thank the Chief Scientist Office for a grant (CZH/4/878), NHS Health Scotland for a supplementary grant (no number), and Information Services Division (ISD) of NHS National Services Scotland and National Records of Scotland for in-house technical support. S.V.K. acknowledges funding from a 10.13039/100003186NRS Senior Clinical Fellowship (SCAF/15/02), the 10.13039/501100000265Medical Research Council (MC_UU_12017/13 & MC_UU_12017/15) and 10.13039/100014589Scottish Government Chief Scientist Office (SPHSU13 & SPHSU15). A.S. is supported by the Farr Institute and Health Data Research UK.

### Competing interests

None declared.

### Author contributions

All authors contributed to the design of the study. G.C. and L.G. drafted the article. R.B. was the principal investigator. L.G. chaired the study group including all the authors. A.D. managed the study. G.C. and M.S. carried out the statistical analysis. All authors commented on the drafts and approved the final version.

### Data sharing

Researchers who wish to access the data should apply to National Records of Scotland (https://www.nrscotland.gov.uk/) and ISD (http://www.isdscotland.org/). They are maintained in a secure environment and governed by ethical and other restrictions on access.

## References

[1] Johnson M., Bhopal R., Ingleby J., Gruer L., Petrova-Benedict R. (2019). A glossary for the first world congress on migration, ethnicity, race and health.

[2] Aldridge R.W., Nellums L.B., Bartlett S., Barr A.L., Patel P., Burns R. (2018). Global patterns of mortality in international migrants: a systematic review and meta-analysis. Lancet.

[3] Bhopal R.S., Gruer L., Cezard G., Douglas A., Steiner M.F.C., Millard A. (2018). Mortality, ethnicity, and country of birth on a national scale, 2001–2013: a retrospective cohort (Scottish Health and Ethnicity Linkage Study). PLoS Med.

[4] Ikram U.Z., Mackenbach J.P., Harding S., Rey G., Bhopal R.S., Regidor E. (2016). All-cause and cause-specific mortality of different migrant populations in Europe. Eur J Epidemiol.

[5] Gruer L. (2018). 1st world congress on migration, ethnicity, race and health: abstract supplement.

[6] Haagsma J.A., Graetz N., Bolliger I., Naghavi M., Higashi H., Mullany E.C. (2016). The global burden of injury: incidence, mortality, disability-adjusted life years and time trends from the Global Burden of Disease study 2013. Inj Prev.

[7] Davis C., Wrigglesworth S. (2018). Unintentional injuries in Scotland.

[8] Deandrea S., Lucenteforte E., Bravi F., Foschi R., La Vecchia C., Negri E. (2010). Review article: risk factors for falls in community-dwelling older people: "a systematic review and meta-analysis". Epidemiology.

[9] Kwan M.M.S., Close J.C., Wong A.K.W., Lord S.R. (2011). Falls incidence, risk factors, and consequences in Chinese older people: a systematic review. J Am Geriatr Soc.

[10] Oliver D., Daly F., Martin F.C., McMurdo M.E. (2004). Risk factors and risk assessment tools for falls in hospital in-patients: a systematic review. Age Ageing.

[11] Hingson R., Howland J. (1987). Alcohol as a risk factor for injury or death resulting from accidental falls: a review of the literature. J Stud Alcohol.

[12] NHS Digital (2019). Statistics of alcohol 2019.

[13] Kyobutungi C., Ronellenfitsch U., Razum O., Becher H. (2006). Mortality from external causes among ethnic German immigrants from former Soviet Union countries, in Germany. Eur J Publ Health.

[14] Norredam M., Olsbjerg M., Petersen J.H., Laursen B., Krasnik A. (2013). Are there differences in injury mortality among refugees and immigrants compared with native-born?. Inj Prev.

[15] Stirbu I., Kunst A., Bos V., van Beeck E.F. (2006). Injury mortality among ethnic minority groups in The Netherlands. J Epidemiol Community.

[16] Han B.H., Ferris R., Blaum C. (2014). Exploring ethnic and racial differences in falls among older adults. J Community Health.

[17] Campos-Outcalt D., Bay C., Dellapena A., Cota M.K. (2003). Motor vehicle crash fatalities by race/ethnicity in Arizona, 1990–96. Inj Prev.

[18] Cercarelli L., Knuiman M. (2002). Trends in road injury hospitalisation rates for Aboriginal and non-Aboriginal people in Western Australia, 1971–97. Inj Prev.

[19] Hosking J., Ameratunga S., Exeter D., Stewart J., Bell A. (2013). Ethnic, socioeconomic and geographical inequalities in road traffic injury rates in the Auckland region. Aust N Z J Publ Health.

[20] Bhopal R., Fischbacher C., Povey C., Chalmers J., Mueller G., Steiner M. (2010). Cohort profile: scottish Health and Ethnicity Linkage Study of 4.65 million people exploring ethnic variations in disease in Scotland. Int J Epidemiol.

[21] Scottish health and ethnicity linkage study website. https://www.ed.ac.uk/usher/scottish-health-ethnicity-linkage.

[22] Bhala N., Cézard G., Ward H.J., Bansal N., Bhopal R. (2016). Ethnic variations in liver-and alcohol-related disease hospitalisations and mortality: the Scottish Health and Ethnicity Linkage study. Alcohol Alcohol.

[23] Bhopal R., Steiner M.F., Cezard G., Bansal N., Fischbacher C., Simpson C.R. (2015). Risk of respiratory hospitalization and death, readmission and subsequent mortality: scottish health and ethnicity linkage study. Eur J Publ Health.

[24] Bhopal R.S., Bansal N., Steiner M., Brewster D.H., Scottish H., Ethnicity Linkage S. (2012). Does the 'Scottish effect' apply to all ethnic groups? All-cancer, lung, colorectal, breast and prostate cancer in the Scottish Health and Ethnicity Linkage Cohort Study. BMJ Open.

[25] Cezard G.I., Bhopal R.S., Ward H.J., Bansal N., Bhala N. (2015). Ethnic variations in upper gastrointestinal hospitalizations and deaths: the scottish health and ethnicity linkage study. Eur J Publ Health.

[26] Gruer L.D., Millard A.D., Williams L.J., Bhopal R.S., Katikireddi S.V., Cézard G.I. (2018). Differences in all-cause hospitalisation by ethnic group: a data linkage cohort study of 4.62 million people in Scotland, 2001–2013. Publ Health.

[27] Katikireddi S.V., Cezard G., Bhopal R.S., Williams L., Douglas A., Millard A. (2018). Assessment of health care, hospital admissions, and mortality by ethnicity: population-based cohort study of health-system performance in Scotland. Lancet Publ Health.

[28] Sheikh A., Steiner M.F., Cezard G., Bansal N., Fischbacher C., Simpson C.R. (2016). Ethnic variations in asthma hospital admission, readmission and death: a retrospective, national cohort study of 4.62 million people in Scotland. BMC Med.

[29] Scottish Government (2017). A fairer Scotland for all: race equality action plan and highlight report 2017-2021.

[30] Fischbacher C., Bhopal R., Povey C., Steiner M., Chalmers J., Mueller G. (2007). Record linked retrospective cohort study of 4.6 million people exploring ethnic variations in disease: myocardial infarction in South Asians. BMC Publ Health.

[31] Fischbacher C.M., Cezard G., Bhopal R.S., Pearce J., Bansal N. (2014). Measures of socioeconomic position are not consistently associated with ethnic differences in cardiovascular disease in Scotland: methods from the Scottish Health and Ethnicity Linkage Study (SHELS). Int J Epidemiol.

[32] Hanlon J.T., Landerman L.R., Fillenbaum G.G., Studenski S. (2002). Falls in African American and white community-dwelling elderly residents. J Gerontol Series A: Biol Sci Med Sci.

[33] Stanaway F.F., Cumming R.G., Naganathan V., Blyth F.M., Handelsman D.J., Le Couteur D.G. (2011). Ethnicity and falls in older men: low rate of falls in Italian-born men in Australia. Age Ageing.

[34] Kwan M.M., Tsang W.W., Lin S.-I., Greenaway M., Close J.C., Lord S.R. (2013). Increased concern is protective for falls in Chinese older people: the chopstix fall risk study. J Gerontol Series A: Biomed Sci Med Sci.

[35] Steinbach R., Edwards P., Green J., Grundy C. (2007). Road safety of london's Black and asian minority ethnic groups. A report to the london road safety unit.

[36] Steinbach R., Edwards P., Green J. (2014). Controlling for exposure changes the relationship between ethnicity, deprivation and injury: an observational study of child pedestrian injury rates in London. Inj Prev.

[37] Steinbach R., Green J., Edwards P., Grundy C. (2010). ‘Race’or place? Explaining ethnic variations in childhood pedestrian injury rates in London. Health Place.

[38] Abdel-Rahman N., Siman-Tov M., Group I.T., Peleg K. (2013). Ethnicity and road traffic injuries: differences between Jewish and Arab children in Israel. Ethn Health.

[39] Smith G.D., Chaturvedi N., Harding S., Nazroo J., Williams R. (2000). Ethnic inequalities in health: a review of UK epidemiological evidence. Crit Publ Health.

[40] Todd C., Ballinger C., Whitehead S. (2007). Reviews of socio-demographic factors related to falls and environmental interventions to prevent falls amongst older people living in the community. http://www.who.int/ageing/projects/3Environmental%20and%20socioeconomic%20risk%20factors%20on%20falls%20pdf.

[41] Jarvis L. (2008). Alcohol consumption in Black and Minority Ethnic groups and recent immigrants in Scotland: current situation on information available.

[42] Transport Scotland (2019). Transport and travel in Scotland. Results from the scottish household survey 2018.

[43] Cycling UK (2019). Cycling UK's Cycling Statistics. Occupation, income, ethnicity and impairment. https://www.cyclinguk.org/statistics.

