# Supplementary material for: Ethnic variations in falls and road traffic injuries resulting in hospitalisation or death in Scotland: the Scottish Health and Ethnicity Linkage Study
Source: Public Health. 2020 May;182:32–8. doi: 10.1016/j.puhe.2020.01.013 (PMC7294220; doi:10.1016/j.puhe.2020.01.013)
Supplement: Multimedia component 1 [file mmc1.docx]

**Ethnic variations in falls and road traffic injuries resulting in hospitalisation or death in Scotland: the Scottish Health and Ethnicity Linkage Study (SHELS)**

**Supplementary Files**

**Supplementary File A**

**Description of the SHELS linkage methods**

Supplementary figure A1, republished from our open-access publication with this edited version of linkage methods,(1) illustrates how record linkage was based on information from three datasets: healthcare records, which include personal identifiers and clinical information; the CHI (Community Health Index) file which contains personal identifiers and the CHI number; and the census file which contains the census number, personal identifiers and details of individuals’ ethnicity (and many other characteristics). The CHI dataset lists everyone in Scotland registered with a general practitioner or eligible for NHS screening services and forms a unique identifier for NHS use. It is estimated that more than 99% of the Scottish population are listed on the CHI. Date of birth, sex, surname (using soundex codes to allow for variations in spelling), forename, address and full postcode, available in Census and CHI, were used to link the census number to the CHI number. For the records deemed to be matches, 73.6% were exact matches on the 6 linking variables. For the remainder, a probability matching process was performed. At this stage, no other variables were in the dataset. The number of people with a valid ethnic group code and the linkage rate, with the estimated total population, is shown in supplementary table A1.

Methods have been developed to identify how false positives occur and what kind of strategies a human checker employs to decide whether a pair match is ‘good’. These decision strategies were built into a ‘partitioning’ computer algorithm. These ‘partitions’ then allow the allocation of effort to the most profitable ‘partitions’ which yield the lowest false-positive and highest true-positive rates. Using methods previously described we estimated an upper limit to the false-positive linkage rate of 0.08%(1).

CHI and the census numbers were encrypted prior to linkage to other datasets. A one-way cryptographic (‘hashing’) algorithm was used to encrypt the CHI number. The census number was encrypted using an algorithm developed by NRS (National Records of Scotland). Once the linkage was completed personal identifying variables (such as names, address, postcode and dates of birth) were removed leaving a file with an encrypted CHI number and its corresponding encrypted census number (look up file).

A census extract containing ethnic group (and limited other data including age, sex and indicators of socio-economic status) was joined to the above look-up file using the encrypted census number. The encrypted census numbers were then discarded leaving ethnicity and other variables from the census, the encrypted CHI number and a newly generated index number unrelated to other numbers for the exclusive use of this project. The relevant parts of the database of hospitalisations and deaths held at ISD were linked via the encrypted CHI numbers. The encrypted CHI was replaced with an unrelated serial number (to keep together the multiple records on the same people), resulting in depersonalised clinical health records carrying census derived ethnicity codes and other relevant census data.

**Figure A1. Overview of Record Linkage Process.**


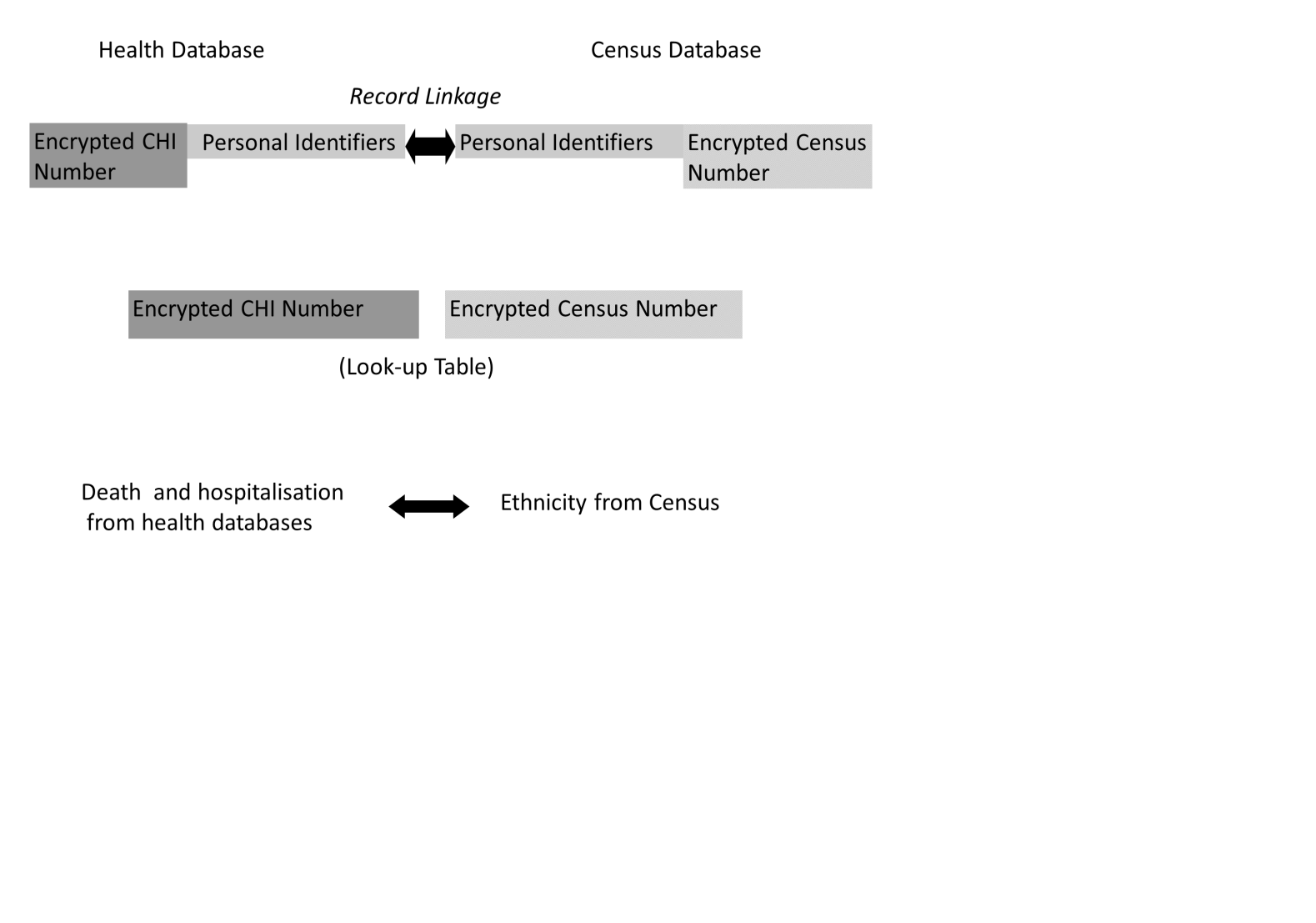


**Table A1. Linkage rates of 2001 Census to Community Health Index (CHI) by ethnic group in relation to total population in census and estimated for Scotland.**

|  | **Ethnicity** | **Number of people completing census** | **% Linked** |
| --- | --- | --- | --- |
| 1 | White Scottish | 4290153 | 95.3 |
| 2 | Other White British | 357788 | 93.6 |
| 3 | White Irish | 47173 | 92.2 |
| 4 | Other White | 74655 | 87.9 |
| 5 | Any mixed background | 12117 | 91.7 |
| 6 | Indian | 13717 | 89.9 |
| 7 | Pakistani | 28538 | 89.8 |
| 8 | Bangladeshi | 1783 | 88.0 |
| 9 | Other South Asian | 5810 | 85.1 |
| 10 | Caribbean | 1659 | 89.5 |
| 11 | African | 4514 | 86.5 |
| 12 | Black Scottish or other Black | 1057 | 89.1 |
| 13 | Chinese | 15115 | 87.4 |
| 14 | Other ethnic group | 8945 | 86.2 |
|  | Total completing census | 4.86 million | 94 |
|  | Total population of Scotland (including estimated numbers) | 5.06 million | 91 |

**Supplementary file B**

**The STROBE/RECORD statement – checklist of items, extended from the STROBE statement, that should be reported in observational studies using routinely collected health data.**

|  | **Item No.** | **STROBE items** | **Location in manuscript where items are reported** | **RECORD items** | **Location in manuscript where items are reported** |
| --- | --- | --- | --- | --- | --- |
| **Title and abstract** | | | | | |
|  | 1 | (a) Indicate the study’s design with a commonly used term in the title or the abstract (b) Provide in the abstract an informative and balanced summary of what was done and what was found | *Abstract* | RECORD 1.1: The type of data used should be specified in the title or abstract. When possible, the name of the databases used should be included.  RECORD 1.2: If applicable, the geographic region and timeframe within which the study took place should be reported in the title or abstract.  RECORD 1.3: If linkage between databases was conducted for the study, this should be clearly stated in the title or abstract. | *Abstract*  *Abstract*  *Title and abstract* |
| **Introduction** | | | | | |
| Background rationale | 2 | Explain the scientific background and rationale for the investigation being reported | *Abstract and introduction* |  |  |
| Objectives | 3 | State specific objectives, including any prespecified hypotheses | *Introduction and methods* |  |  |
| **Methods** | | | | | |
| Study Design | 4 | Present key elements of study design early in the paper | *Methods* |  |  |
| Setting | 5 | Describe the setting, locations, and relevant dates, including periods of recruitment, exposure, follow-up, and data collection | *Methods, and supplementary file A.* |  |  |
| Participants | 6 | *(a) Cohort study* - Give the eligibility criteria, and the sources and methods of selection of participants. Describe methods of follow-up  *Case-control study* - Give the eligibility criteria, and the sources and methods of case ascertainment and control selection. Give the rationale for the choice of cases and controls  *Cross-sectional study* - Give the eligibility criteria, and the sources and methods of selection of participants  *(b) Cohort study* - For matched studies, give matching criteria and number of exposed and unexposed  *Case-control study* - For matched studies, give matching criteria and the number of controls per case | *Methods, citing and supplementary file A.* | RECORD 6.1: The methods of study population selection (such as codes or algorithms used to identify subjects) should be listed in detail. If this is not possible, an explanation should be provided.  RECORD 6.2: Any validation studies of the codes or algorithms used to select the population should be referenced. If validation was conducted for this study and not published elsewhere, detailed methods and results should be provided.  RECORD 6.3: If the study involved linkage of databases, consider use of a flow diagram or other graphical display to demonstrate the data linkage process, including the number of individuals with linked data at each stage. | *ICD10 codes for case selection given in detail in Methods.*  *Not done*  *Flow diagram in Supplementary file A* |
| Variables | 7 | Clearly define all outcomes, exposures, predictors, potential confounders, and effect modifiers. Give diagnostic criteria, if applicable. | *Methods* | RECORD 7.1: A complete list of codes and algorithms used to classify exposures, outcomes, confounders, and effect modifiers should be provided. If these cannot be reported, an explanation should be provided. | *Methods,* |
| Data sources/ measurement | 8 | For each variable of interest, give sources of data and details of methods of assessment (measurement).  Describe comparability of assessment methods if there is more than one group | *Methods* |  |  |
| Bias | 9 | Describe any efforts to address potential sources of bias | *Methods. We note the high linkage rate for all ethnic groups* *(>85%) in the study.*  *Discussion: we note we could not exclude some denominator bias as some members of the cohort could have left the UK without our knowledge.* |  |  |
| Study size | 10 | Explain how the study size was arrived at | *Methods* |  |  |
| Quantitative variables | 11 | Explain how quantitative variables were handled in the analyses. If applicable, describe which groupings were chosen, and why | *Not applicable* |  |  |
| Statistical methods | 12 | (a) Describe all statistical methods, including those used to control for confounding  (b) Describe any methods used to examine subgroups and interactions  (c) Explain how missing data were addressed  (d) *Cohort study* - If applicable, explain how loss to follow-up was addressed  *Case-control study* - If applicable, explain how matching of cases and controls was addressed  *Cross-sectional study* - If applicable, describe analytical methods taking account of sampling strategy  (e) Describe any sensitivity analyses | *Methods*  *Methods: we corrected for death and departure from NHS Scotland to elsewhere in the United Kingdom (UK).* |  |  |
| Data access and cleaning methods |  | .. |  | RECORD 12.1: Authors should describe the extent to which the investigators had access to the database population used to create the study population.  RECORD 12.2: Authors should provide information on the data cleaning methods used in the study. | *Methods*  *Supplementary file A.* |
| Linkage |  | .. |  | RECORD 12.3: State whether the study included person-level, institutional-level, or other data linkage across two or more databases. The methods of linkage and methods of linkage quality evaluation should be provided. | *Supplementary file A.* |
| **Results** | | | | | |
| Participants | 13 | (a) Report the numbers of individuals at each stage of the study (*e.g.*, numbers potentially eligible, examined for eligibility, confirmed eligible, included in the study, completing follow-up, and analysed)  (b) Give reasons for non-participation at each stage.  (c) Consider use of a flow diagram | *As rates were calculated using person years at risk after correction, we did not report numbers of individuals during the course of the follow-up period.* | RECORD 13.1: Describe in detail the selection of the persons included in the study (*i.e.,* study population selection) including filtering based on data quality, data availability and linkage. The selection of included persons can be described in the text and/or by means of the study flow diagram. | *Methods and supplementary file A.* |
| Descriptive data | 14 | (a) Give characteristics of study participants (*e.g.*, demographic, clinical, social) and information on exposures and potential confounders  (b) Indicate the number of participants with missing data for each variable of interest  (c) *Cohort study* - summarise follow-up time (*e.g.*, average and total amount) | *Results text and Table 1.*  *Participants were included in the cohort only if all the variables of interest were recorded.* |  |  |
| Outcome data | 15 | *Cohort study* - Report numbers of outcome events or summary measures over time  *Case-control study* - Report numbers in each exposure category, or summary measures of exposure  *Cross-sectional study* - Report numbers of outcome events or summary measures | Results |  |  |
| Main results | 16 | (a) Give unadjusted estimates and, if applicable, confounder-adjusted estimates and their precision (e.g., 95% confidence interval). Make clear which confounders were adjusted for and why they were included  (b) Report category boundaries when continuous variables were categorized  (c) If relevant, consider translating estimates of relative risk into absolute risk for a meaningful time period | *We give age-adjusted and confounder adjusted estimates.* |  |  |
| Other analyses | 17 | Report other analyses done—e.g., analyses of subgroups and interactions, and sensitivity analyses | *Other analyses not done* |  |  |
| **Discussion** | | | | | |
| Key results | 18 | Summarise key results with reference to study objectives | *1st paragraph* |  |  |
| Limitations | 19 | Discuss limitations of the study, taking into account sources of potential bias or imprecision. Discuss both direction and magnitude of any potential bias | *2^nd^ paragraph* | RECORD 19.1: Discuss the implications of using data that were not created or collected to answer the specific research question(s). Include discussion of misclassification bias, unmeasured confounding, missing data, and changing eligibility over time, as they pertain to the study being reported. | *Possible biases discussed due to possibly different rates of loss to follow-up or different likelihood of attendance at hospital.* |
| Interpretation | 20 | Give a cautious overall interpretation of results considering objectives, limitations, multiplicity of analyses, results from similar studies, and other relevant evidence | *Done* |  |  |
| Generalisability | 21 | Discuss the generalisability (external validity) of the study results | *Due to the unique nature of each country ethnic mix, generalizability is limited.* |  |  |
| **Other Information** | | | | | |
| Funding | 22 | Give the source of funding and the role of the funders for the present study and, if applicable, for the original study on which the present article is based | *The sources of funding are given in the acknowledgements* |  |  |
| Accessibility of protocol, raw data, and programming code |  | .. |  | RECORD 22.1: Authors should provide information on how to access any supplemental information such as the study protocol, raw data, or programming code. | *The study protocol is referenced and available. How to access the data is explained at the end of the paper.* |

*Reference: Benchimol EI, Smeeth L, Guttmann A, Harron K, Moher D, Petersen I, Sørensen HT, von Elm E, Langan SM, the RECORD Working Committee. The REporting of studies Conducted using Observational Routinely-collected health Data (RECORD) Statement. *PLoS Medicine* 2015; in press*Checklist is protected under Creative Commons Attribution ([CC BY](http://creativecommons.org/licenses/by/4.0/)) license.

**Supplementary File C**

**Table C1. Age adjusted rates per 100,000 person years at risk (PY) and risk ratios (RR) for hospitalisations or deaths due to injury of car occupants (V40-V49) by sex and ethnic group. RRs are adjusted for age and additionally for SES, UK/RoI-born and both, with 95% confidence intervals.**

| Sex and ethnic group | Cases | PY | Age-adjusted Poisson rates (per 100,000 PY) | Age adjusted  RR (95% CI) | Age + SES  RR (95% CI) | Age + UK/RoI-born  RR (95% CI) | Age + SES +UK/RoI-born  RR (95% CI) |
| --- | --- | --- | --- | --- | --- | --- | --- |
| **MALES** |  |  |  |  |  |  |  |
| White Scottish | 9205 | 21179755 | 44 | 100 | 100 | 100 | 100 |
| Other White British | 690 | 1571080 | 56 | 132 (115, 153) | 124 (108, 143) | 134 (116, 155) | 125 (108, 144) |
| White Irish | 50 | 202190 | 29 | 68 (52, 90) | 69 (53, 91) | 69 (52, 91) | 70 (53, 92) |
| Other White | 90 | 278515 | 41 | 96 (76, 121) | 94 (74, 119) | 95 (75, 120) | 94 (74, 119) |
| Any mixed background | 15 | 56265 | 21 | 49 (30, 82) | 50 (30, 83) | 50 (30, 82) | 50 (30, 83) |
| Indian | 25 | 65945 | 49 | 115 (77, 171) | 111 (75, 165) | 115 (77, 171) | 111 (75, 165) |
| Pakistani | 65 | 146430 | 57 | 135 (105, 174) | 132 (103, 171) | 135 (104, 173) | 132 (103, 170) |
| Other South Asian | 15 | 35500 | 56 | 132 (85, 207) | 136 (87, 212) | 132 (84, 206) | 136 (87, 212) |
| African origin | 10 | 32160 | 34 | 80 (44, 145) | 84 (47, 153) | 80 (44, 145) | 84 (46, 153) |
| Chinese | 20 | 68685 | 59 | 140 (92, 212) | 139 (92, 211) | 139 (92, 210) | 139 (92, 210) |
| **FEMALES** |  |  |  |  |  |  |  |
| White Scottish | 6955 | 22581190 | 31 | 100 | 100 | 100 | 100 |
| Other White British | 590 | 1644435 | 37 | 124 (105, 147) | 114 (96, 134) | 128 (108, 152) | 116 (97, 138) |
| White Irish | 45 | 216905 | 22 | 73 (54, 98) | 74 (55, 100) | 75 (56, 102) | 76 (56, 103) |
| Other White | 100 | 319915 | 38 | 128 (101, 161) | 121 (96, 152) | 124 (98, 156) | 118 (94, 149) |
| Any mixed background | 10 | 59970 | 19 | 62 (36, 107) | 63 (37, 109) | 63 (36, 109) | 64 (37, 110) |
| Indian | 10 | 59925 | 33 | 109 (65, 185) | 108 (64, 182) | 108 (64, 183) | 107 (63, 181) |
| Pakistani | 35 | 143940 | 32 | 106 (77, 147) | 110 (80, 152) | 106 (77, 147) | 110 (80, 152) |
| Other South Asian | 10 | 28610 | 38 | 127 (70, 232) | 125 (69, 228) | 127 (70, 231) | 125 (69, 227) |
| African origin | 10 | 28590 | 34 | 114 (59, 221) | 122 (63, 235) | 113 (58, 219) | 121 (63, 233) |
| Chinese | 15 | 68010 | 41 | 137 (84, 222) | 139 (85, 225) | 134 (82, 217) | 137 (84, 222) |

| Sex and ethnic group | Cases | PY | Age-adjusted Poisson rates (per 100,000 PY) | Age adjusted  RR (95% CI) | Age + SES  RR (95% CI) | Age + UK/RoI-born  RR (95% CI) | Age + SES + UK/RoI-born  RR (95% CI) |
| --- | --- | --- | --- | --- | --- | --- | --- |
| **MALES** |  |  |  |  |  |  |  |
| White Scottish | 7525 | 21110105 | 36 | 100 | 100 | 100 | 100 |
| Other White British | 600 | 1563845 | 68 | 195 (165, 231) | 181 (153, 214) | 206 (173, 244) | 189 (159, 224) |
| White Irish | 45 | 200745 | 41 | 119 (88, 161) | 122 (90, 166) | 127 (93, 172) | 128 (95, 175) |
| Other White | 90 | 276960 | 48 | 138 (108, 176) | 130 (102, 166) | 129 (100, 166) | 123 (96, 159) |
| Any mixed background | 30 | 56230 | 38 | 110 (76, 160) | 108 (74, 157) | 113 (78, 165) | 110 (76, 161) |
| Indian | 10 | 65795 | 19 | 55 (30, 101) | 53 (29, 97) | 56 (31, 102) | 53 (29, 98) |
| Pakistani | 25 | 146250 | 20 | 56 (38, 84) | 62 (41, 93) | 57 (38, 85) | 62 (41, 93) |
| Other South Asian | 10 | 35455 | 42 | 120 (71, 202) | 122 (72, 206) | 117 (69, 198) | 120 (71, 202) |
| African origin | 10 | 32115 | 35 | 99 (51, 194) | 100 (51, 196) | 96 (49, 188) | 98 (50, 191) |
| Chinese | 10 | 68630 | 30 | 87 (49, 155) | 88 (49, 157) | 85 (47, 151) | 86 (48, 154) |
| **FEMALES** |  |  |  |  |  |  |  |
| White Scottish | 2035 | 22450550 | 9 | 100 | 100 | 100 | 100 |
| Other White British | 180 | 1632520 | 22 | 245 (163, 367) | 218 (145, 328) | 273 (182, 410) | 240 (160, 360) |
| White Irish | 10 | 213960 | 12 | 131 (68, 253) | 131 (68, 252) | 150 (78, 291) | 147 (76, 284) |
| Other White | 40 | 318230 | 25 | 281 (174, 452) | 254 (158, 409) | 241 (150, 388) | 222 (138, 357) |
| Any mixed background | 10 | 59920 | 15 | 172 (83, 354) | 171 (83, 351) | 188 (91, 388) | 184 (89, 379) |
| Indian | . | 59745 | . | . | . | . | . |
| Pakistani | . | 143710 | . | . | . | . | . |
| Other South Asian | . | 28550 | . | . | . | . | . |
| African origin | . | 28510 | . | . | . | . | . |
| Chinese | . | 67855 | . | . | . | . | . |

**Table C2. Age adjusted rates per 100,000 person years at risk (PY) and risk ratios (RR) for hospitalisations or deaths due to injuries of cyclists (V10-V19) by sex and ethnic group. RRs are adjusted for age and additionally for SES, UK/RoI-born and both, with 95% confidence intervals.**

| Sex and ethnic group | Cases | PY | Age-adjusted Poisson rates (per 100,000 PY) | Age adjusted  RR (95% CI ) | Age + SES  RR (95% CI) | Age + UK/RoI-born  RR (95% CI) | Age + SES + UK/RoI-born  RR (95% CI) |
| --- | --- | --- | --- | --- | --- | --- | --- |
| **MALES** |  |  |  |  |  |  |  |
| White Scottish | 4385 | 21173185 | 21 | 100 | 100 | 100 | 100 |
| Other White British | 185 | 1570410 | 16 | 79 (62, 100) | 85 (67, 108) | 78 (61, 99) | 85 (67, 108) |
| White Irish | 45 | 202090 | 28 | 138 (96, 197) | 131 (92, 188) | 136 (95, 194) | 130 (91, 187) |
| Other White | 45 | 278450 | 20 | 98 (70, 137) | 101 (72, 142) | 100 (70, 141) | 102 (72, 144) |
| Any mixed background | 15 | 56265 | 22 | 108 (65, 180) | 106 (64, 177) | 108 (65, 179) | 106 (64, 176) |
| Indian | 10 | 65935 | 12 | 58 (27, 122) | 63 (30, 133) | 58 (27, 122) | 63 (30, 133) |
| Pakistani | 30 | 146375 | 23 | 111 (77, 161) | 111 (77, 161) | 111 (77, 162) | 111 (77, 161) |
| Other South Asian | 10 | 35485 | 29 | 142 (73, 278) | 139 (71, 271) | 143 (73, 278) | 139 (71, 272) |
| African origin | 10 | 32160 | 32 | 160 (82, 313) | 148 (75, 290) | 161 (82, 315) | 148 (75, 291) |
| Chinese | 10 | 68665 | 30 | 149 (81, 273) | 153 (83, 280) | 150 (82, 275) | 153 (83, 281) |
| **FEMALES** |  |  |  |  |  |  |  |
| White Scottish | 2445 | 22556770 | 11 | 100 | 100 | 100 | 100 |
| Other White British | 105 | 1642245 | 8 | 71 (52, 97) | 77 (56, 106) | 73 (52, 102) | 80 (58, 112) |
| White Irish | 20 | 216595 | 9 | 88 (53, 147) | 84 (50, 140) | 91 (54, 154) | 88 (52, 149) |
| Other White | 15 | 319675 | 6 | 54 (32, 93) | 57 (33, 98) | 52 (30, 91) | 54 (31, 95) |
| Any mixed background | 10 | 59965 | 10 | 93 (46, 190) | 91 (45, 185) | 95 (47, 194) | 94 (46, 191) |
| Indian | . | 59885 | . | . | . | . | . |
| Pakistani | 15 | 143895 | 12 | 112 (65, 192) | 110 (64, 189) | 112 (66, 193) | 110 (64, 189) |
| Other South Asian | . | 28595 | . | . | . | . | . |
| African origin | 10 | 28585 | 25 | 234 (110, 497) | 218 (103, 462) | 232 (110, 492) | 215 (102, 456) |
| Chinese | 10 | 67995 | 22 | 205 (101, 416) | 209 (103, 423) | 200 (98, 406) | 201 (99, 408) |

**Table C3. Age adjusted rates per 100,000 person years at risk (PY) and risk ratios (RR) for hospitalisation or death due to injuries of pedestrians (V00-V09) by sex and ethnic group. RRs are adjusted for age and additionally for SES, UK/RoI-born and both, with 95% confidence intervals.**

**Table C4. Age adjusted rates per 100,000 person years at risk (PY) and risk ratios (RR) for hospitalisations or deaths due to injuries of motorcyclists (V20-V29) by sex and ethnic group. RRs are adjusted for age and additionally for SES, UK/RoI-born and both, with 95% confidence intervals**

| Sex and ethnic group | Cases | PY | Age-adjusted Poisson rates (per 100,000 PY) | Age adjusted  RR (95% CI ) | Age + SES  RR (95% CI) | Age + UK/RoI-born  RR (95% CI) | Age + SES + UK/RoI-born  RR (95% CI) |
| --- | --- | --- | --- | --- | --- | --- | --- |
| **MALES** |  |  |  |  |  |  |  |
| White Scottish | 5450 | 21179755 | 26 | 100 | 100 | 100 | 100 |
| Other White British | 410 | 1571080 | 61 | 242 (185-316) | 226 (173-296) | 246 (186-325) | 229 (173-303) |
| White Irish | 20 | 202190 | 22 | 89 (56-140) | 90 (57-142) | 90 (57-144) | 91 (57-146) |
| Other White | 65 | 278515 | 50 | 199 (140-283) | 197 (138-281) | 196 (138-278) | 195 (137-277) |
| Any mixed background | 10 | 56265 | 28 | 110 (60-203) | 113 (61-208) | 111 (60-205) | 114 (62-209) |
| Indian | . | 65945 | . | . | . | . | . |
| Pakistani | 15 | 146430 | 19 | 75 (42-134) | 73 (41-131) | 75 (42-134) | 73 (41-130) |
| Other South Asian | 10 | 35500 | 47 | 188 (90-394) | 194 (92-407) | 187 (89-391) | 193 (92-405) |
| African origin | . | 32160 | . | . | . | . | . |
| Chinese | . | 68685 | . | . | . | . | . |
| **FEMALES** |  |  |  |  |  |  |  |
| White Scottish | 520 | 22556770 | 2 | 100 | 100 | 100 | 100 |
| Other White British | 65 | 1642245 | 3 | 135 (83-218) | 123 (76-200) | 143 (83-245) | 129 (75-222) |
| White Irish | . | 216595 | . | . | . | . | . |
| Other White | 10 | 319675 | 3 | 127 (59-273) | 119 (56-255) | 120 (53-273) | 113 (50-258) |
| Any mixed background | . | 59965 | . | . | . | . | . |
| Indian | . | 59745 | . | . | . | . | . |
| Pakistani | . | 143710 | . | . | . | . | . |
| Other South Asian | . | 28550 | . | . | . | . | . |
| African origin | . | 28510 | . | . | . | . | . |
| Chinese | . | 67855 | . | . | . | . | . |
